# Modelling and Microstructural Characterization of Sintered Metallic Porous Materials

**DOI:** 10.3390/ma9070567

**Published:** 2016-07-12

**Authors:** Wojciech Depczynski, Robert Kazala, Krzysztof Ludwinek, Katarzyna Jedynak

**Affiliations:** 1 Faculty of Mechatronics and Mechanical Engineering, Kielce University of Technology, Al. Tysiaclecia P.P. 7, Kielce 25-314, Poland; 2 Faculty of Electrical Engineering, Automatic Control and Computer Science, Kielce University of Technology, Al. Tysiaclecia P.P. 7, Kielce 25-314, Poland; rkazala@tu.kielce.pl (R.K.); k.ludwinek@tu.kielce.pl (K.L.); 3 Institute of Chemistry, Jan Kochanowski University in Kielce, Swietokrzyska 15G, Kielce 25-406, Poland; kjedynak@ujk.edu.pl

**Keywords:** metallic porous materials, metal foam, sintering, diffusion bridges, modelling of porous materials

## Abstract

This paper presents selected characteristics of the metallic porous materials produced by the sintering of metal powders. The authors focus on materials produced from the iron powder (Fe) of ASC 100.29 and Distaloy SE. ASC 100.29 is formed by atomization and has a characteristic morphology. It consists of spherical particles of different sizes forming agglomerates. Distaloy SE is also based on the sponge-iron. The porous material is prepared using the patented method of sintering the mixture of iron powder ASC 100.29, Fe(III) oxide, Distaloy SE and Fe(III) oxide in the reducing atmosphere of dissociated ammonia. As a result, the materials with open pores of micrometer sizes are obtained. The pores are formed between iron particles bonded by diffusion bridges. The modelling of porous materials containing diffusion bridges that allows for three-dimensional (3D) imaging is presented.

## 1. Introduction

The metallic porous materials are a relatively new class of engineering materials, which have been widely researched due to their innovative properties. The available literature provides a broad review of issues related to this field of research [1,2,3,4]. The increase in interest in porous materials is caused by the fact that a porous structure is present in living organisms, for example in the morphology of wood or human bone. The porous structure is characterized by an unusual combination of two properties: high stiffness and minimum weight [1,4].

Highly porous open-cell materials based on various metals and alloys are of increasing interest as they combine structural and functional properties [1,3]. Open-celled metallic foams with their specific structural properties are attractive candidates for a wide range of applications in the field of catalyst supports, process, and energy technologies [1,3,5]. Early attempts to obtain a porous structure were based on the knowledge of foaming polymers, where the blown gas was used as a foaming agent [1,2,3]. Another method focused on the generation of the cell structure by means of granules, which were introduced into the liquid metal or into the casting mould [1,6]. The elements made of these materials have been successfully used in the aerospace, automotive and armaments industries [7].

Although iron alloys are the most commonly used construction materials, the porous materials obtained from them have not been widely applied. This may be caused by the problems with the availability of steel foams on the market or the insufficient number of proposed applications. The research conducted over the last 15–20 years has shown that metal foams, for the purpose of laboratory testing, can be produced on an Fe base. Thus, it is possible to study their properties at different angles [3]. Metal foams that are currently developed and manufactured for industrial use are based mainly on Al, Cu and Fe [8,9,10].

Some technologies are useful for a wide group of materials as described in [11]. Another approach is presented in [12]. The authors obtained the material of predetermined properties by solid-state sintering of 304 stainless steel powders and 304 short stainless steel fibres. The bindings between arbitrary conventional materials and foams are formed by preparing the foam on a metal substrate [10], by means of laser welding techniques [13], or more frequently by the friction stir welding technique [14]. When materials which are distant from each other in the electrochemical series of metals are bonded, the problem of electrochemical corrosion arises as described in [15].

In the search for more efficient and simpler methods, we have proposed a novel low-cost technology for the production of foams from metals, alloys and intermetallics. Metal foam is formed by a typical base metal, for example Cu or Fe. In the experiment, the produced metal foams are tested by the reduction of metal oxides during sintering. The mixture is sintered in a dissociated ammonia atmosphere. The Fe foam is prepared according to the method described in [16]. This allows for the formation of irregular cellular structures with open or closed pores. The range of the porosity strongly depends on the materials used—the particle size and the type of particulate material. However, porosity is strongly affected by the ratio between the quantity of metal oxide powder and the amount of matrix metal powder, which forms a basic structure of the produced sinter [10]. Sinters are widely used, for example, as heat exchangers, filters or catalysts. The foam material can be stacked and co-sintered with top layers to form sandwich structures.

The modelling of structural elements has been applied in engineering practice for a long time. Advanced design systems commonly use the finite element method (FEM) or finite element analysis (FEA) modelling to solve design problems [17,18,19,20]. While for materials with continuous digital structure models are commonly available and reliable, there exist many problems relating to the modelling of porous materials. The pores are formed by the physical-chemical reaction resulting from the production method. Their geometry and mechanical properties are therefore observed in the range of characteristics of the formation process. In the literature [21,22,23], models are based on replacing the actual pore geometry with a simplified shape such as polyhedral solid or spatial structure of the rod. These models allow for linking the physical properties of the materials forming the porous structure with the construction of the cell. However, their shape as represented by the models is a considerable simplification of the actual one.

The model presented in [23] allows for a description with a good approximation. However, in analytical modelling, mathematical descriptions of diffusion bridges are very complex [24,25,26]. The example is the model of the diffusion bridge presented in [23]. The model is described by complex partial differential equations describing interaction forces between particles and mass transport during the diffusion process. Such a method of modelling hinders the application of diffusion bridges in modelling and the simulation of materials of larger volume. In our paper, a different approach to solve the problem of modelling the spatial structure of diffusion bridges is proposed.

The main objective of our paper is to present a proposal of an approach to solve the problem of modelling the spatial structure of diffusion bridges. One of the solutions is the application of metaball objects used in computer graphics to model organic objects [27,28]. We have assessed that this strategy of modelling can be applied to technology for the preparation of metallic porous materials.

## 2. Results

**Figure 1:**
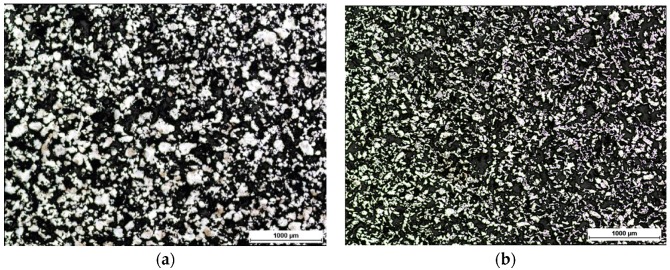
Microstructure of metallic foams (50× magnification) (**a**) ASC 100.29; (**b**) Distaloy SE imaged by optical microscopy.

**Figure 2:**
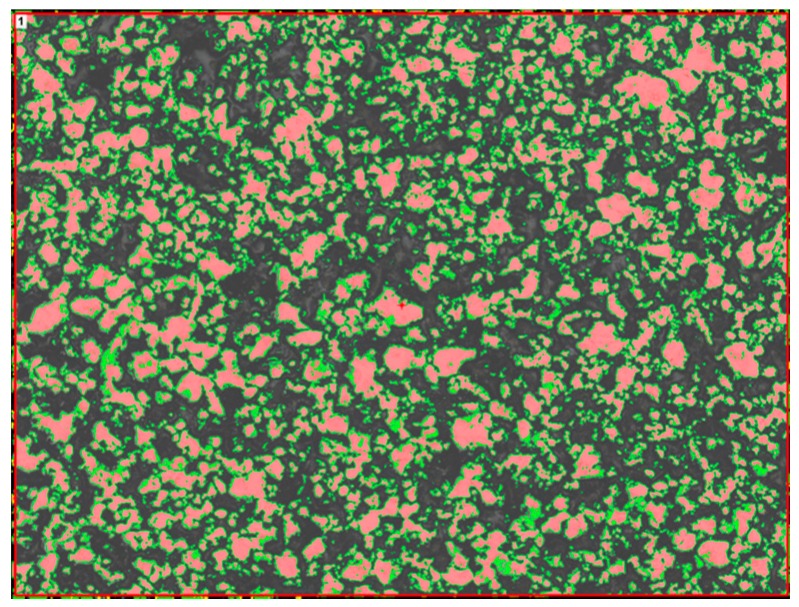
Porosity measurement of ASC 100.29 sample made by optical microscopy and NIS 4.20 software (50× magnification).

The microstructures of a porous metal foam from ASC 100.29 and Distaloy SE were observed using an optical microscope from Nikon MA200 (Tokyo, Japan). The view of ASC 100.29 and Distaloy SE obtained by means of optical microscopy is shown in Figure 1. The unetched samples were analysed. The obtained figures present open porosity and grain-to-grain connections. Figure 2 shows the measurement of porosity on the cross-section by means of the imaging analysis system NIS 4.20. The relative volume of particles was determined in accordance with the Cavalieri-Hacquert principle and the result of the porous metal foam from ASC 100.29 showed a porosity of 62% with bulk density. The same was done for the material obtained from Distaloy SE powder and the result was 75%. The difference in porosity between ASC 100.29 and Distaloy SE is 13%. It is caused by a different creation of diffusion bridges in these materials. The chemical composition and shape of the sintered material particles affect the creation of the diffusion bridges.

**Figure 3:**
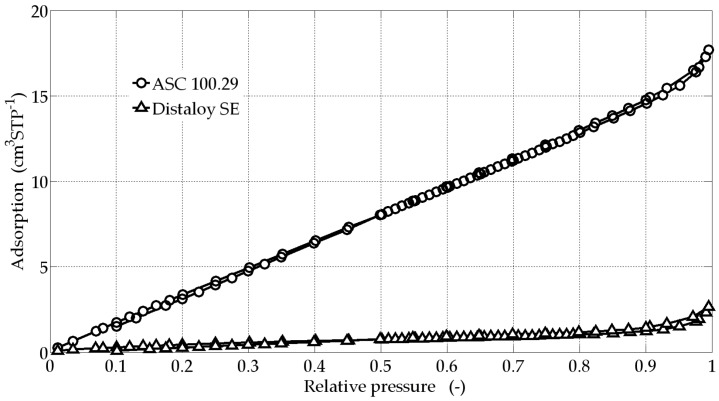
Adsorption isotherms of the samples (Figure 1) tested with LN_2_ at 77 K.

The materials were tested to determine the type of pores and the porosity of the samples. In order to obtain the above-mentioned parameters, the computational methods based on the BET model S_BET_ (Brunauer-Emmett-Teller) were used. Figure 3 shows the characteristics of the adsorption isotherms of the samples tested with LN_2_ at 77 K.

**Figure 4:**
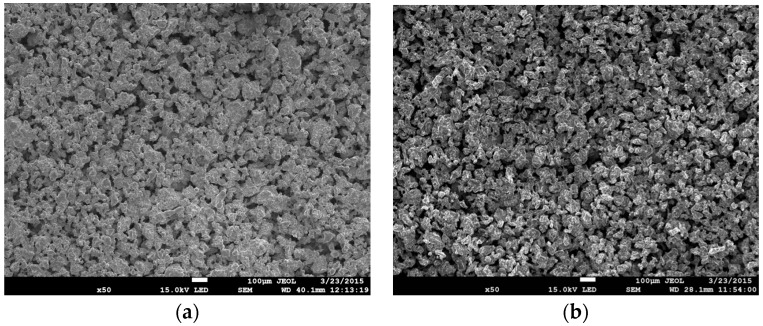
SEM images of foam surface made of sintered (50× magnification): (**a**) ASC 100.29; (**b**) Distaloy SE.

Figure 4 shows SEM microphotographs of sintered foam surfaces of ASC 100.29 (Figure 4a) and Distaloy SE (Figure 4b), imaged by a JEOL JSM 7100F scanning electron microscope (JEOL Musashino, Akishima, Tokyo, Japan). The depth of the field obtained by SEM enables us to assess the complex structure of the foam with visible parent material and voids forming the porosity.

**Figure 5:**
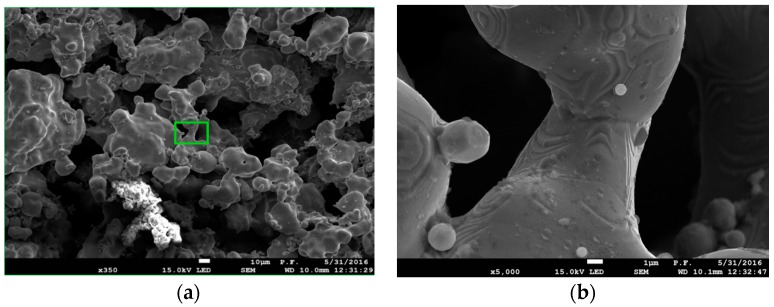
SEM images of ASC 100.29: (**a**) 350× magnification with visible parent material and voids forming porosity with the magnified spot marked; (**b**) 5000× magnification with visible diffusion bridge between two grains of parent material.

**Figure 6:**
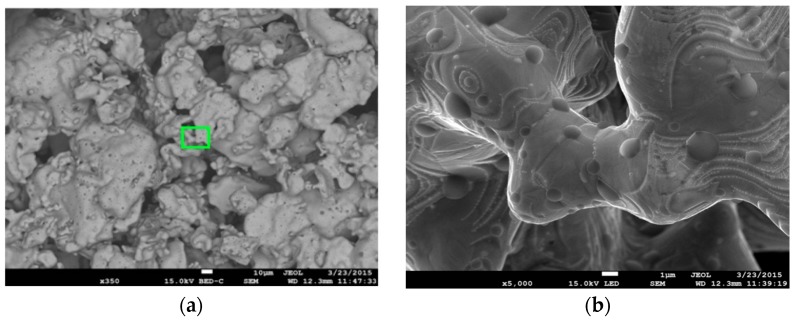
SEM images of Distaloy SE: (**a**) 350× magnification (BSE) with visible parent material and voids forming porosity with the magnified spot marked; (**b**) 5000× magnification (SE) with visible diffusion bridge between two grains of parent material.

Figure 5 and Figure 6 present microstructures of ASC 100.29 metallic foam (350× magnification) with visible parent material and voids forming the porosity and the foam obtained from Distaloy SE with a visible diffusion bridge between two grains of parent material (5000× magnification).

**Figure 7:**
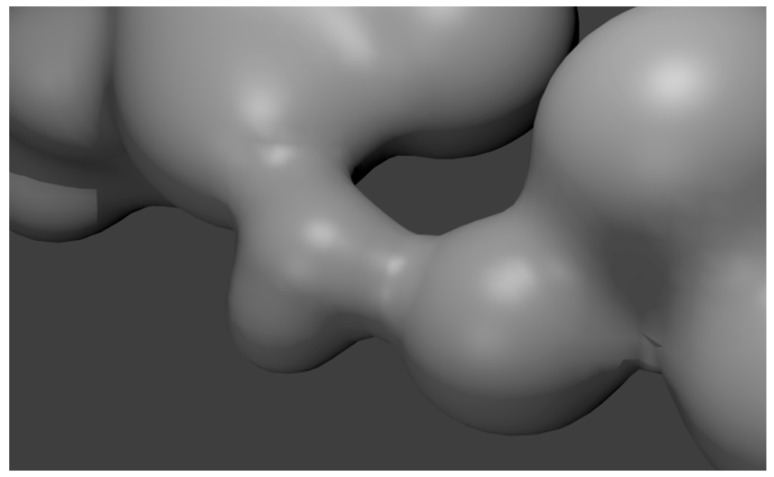
Model of bridge structure corresponding to the real structure shown in Figure 6.

To generate the model structures of diffusion bridges, the metaball objects are implemented in the Blender environment [29]. The sample model of the structure corresponding to the actual structure of the bridge (Figure 6b) is shown in Figure 7.

The sieve analysis of ASC 100.29 showing the percentage of different grain sizes is presented in Table 1. The distributions are determined on the basis of the data used in the production process of porous materials.

**Figure 8:**
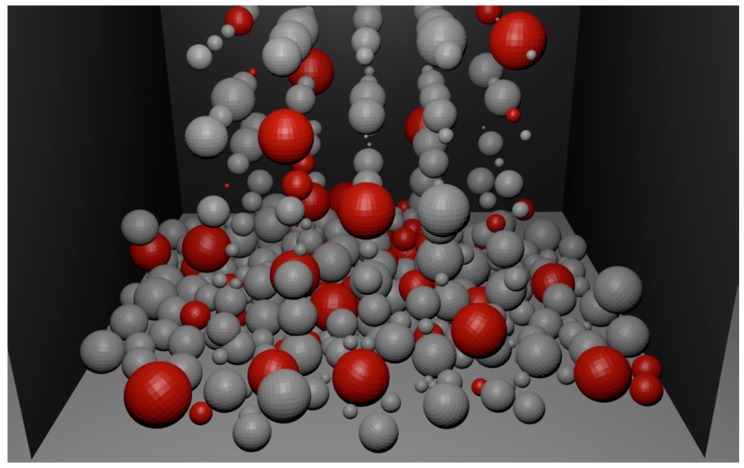
The process of filling the container with porous material.

**Figure 9:**
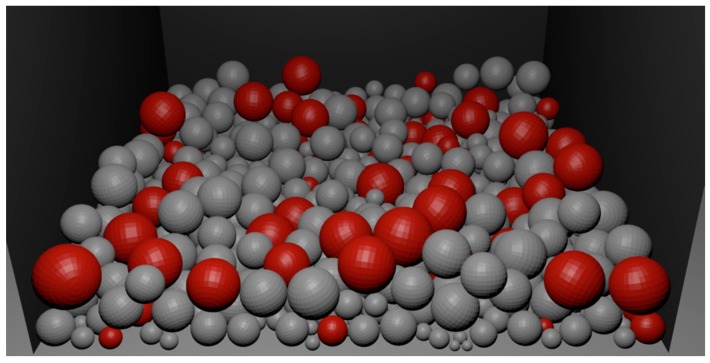
The container filled with porous material.

**Figure 10:**
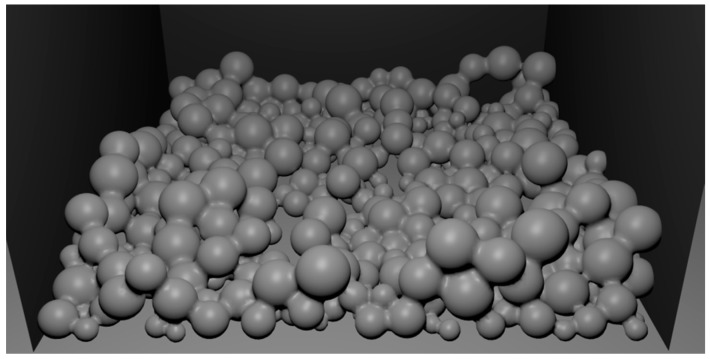
Porous material with newly formed porous bridges.

Figure 8, Figure 9 and Figure 10 present the process of filling a container with a batch of porous material. The initial state of filling is shown in Figure 8. Figure 9 presents the filled container with a batch of porous material. The uneven distribution of iron particles and oxides is visible. After filling the container (Figure 9) the sintering is simulated, as described above, to eliminate the iron oxide particles and form diffusion bridges. In order to imitate the actual spatial configuration, the modelling of diffusion bridges shown in Figure 7 is used in the simulation. The obtained porous material is shown in Figure 10.

It is seen in Figure 10 that diffusion bridges connecting the individual particles of the batch do not have a predetermined fixed direction. Thus, the developed model properly reflects the spatial configuration of the bridges in the actual porous material (Figure 6b).

On the basis of the presented modelling method and simulation results (Figure 7), it can be concluded that it is possible to observe the morphological structure of the elements with complex spatial configurations and obtain the simulation results close to the ones obtained for actual elements.

The applied test methods were used to determine the initial parameters for the modelling process. By obtaining results concerning the morphology of the real materials’ structures, the authors have created very realistic, geometric models corresponding to the real structures.

## 3. Discussion

In previous studies, most of the metallic foams based on Fe using gases generated by the reduction of iron oxide during the process of metal melting [23], or other types of foaming agent during sintering [30,31], with the structure of considerably smaller pores were obtained. The distribution of the pore volume was also much narrower. A similar distribution but for a much larger pore volume was obtained in [32] using an organic foam precursor.

Also, metallic porous materials produced nowadays have different structures and mechanical properties [33] depending on both the type of batch and the sintering method. In order to assess these properties, it is necessary to perform the test on samples of the material obtained. The tests are mostly conducted on samples of standard sizes. When the details of the porous materials have complex configurations of spatial elements, the results of standard tests may be difficult to implement [34]. In this case, it is necessary to conduct experimental tests on the samples of the details in order to obtain mechanical properties. This is, however, expensive and time-consuming, since a large number of samples have to be tested to obtain the required mechanical properties.

Basing upon the tests of structural and mechanical properties, it is possible to determine the properties of the porous material and its spatial configuration. The results can be used to develop the simulation model of a porous material showing its spatial configuration [35]. Further, after the course of studies of metaball modeling has been developed, FEM methods are likely to be used. Due to the highly diverse geometric structures of materials, an analytical description of their mechanical properties is unfeasible.

The simulation models that are currently used are usually based on a simplified structure of the material of regular shape [20,23] with no regard to diffusion bridges. In the literature, different ways of modelling diffusion bridges can be found [23,26,36,37]. However, these methods are not effective in the computer simulation of complex structures with a large amount of batch grains of different sizes. The Blender environment enables generating the geometry of batch particles and their visualization [38]. In order to illustrate the different stages of a porous material’s production, a collection of particles of random size is modelled. Next, physical modelling is used to fill the container with a porous material.

To model porous materials containing diffusion bridges, the authors have employed a specialized library generating objects of metaball type [29]. Such objects are used to model organic structures in computer graphics. On the basis of the analysis, the authors have observed that the mechanism of linking objects of metaball type is very similar to the phenomena taking place when the diffusion bridges are formed in real materials with porous structures (with metallic powders as the base). The SEM image of the diffusion bridges (Figure 6b) compared to the model shown in Figure 7 appear to be very similar, almost identical, which indicates the accuracy of the adopted modelling method (metaball objects). A more complete verification of generated models can be performed by comparing it to the reconstruction of the structures obtained by the μCT technique [39].

To construct the simulation model of porous materials with an arbitrary grain structure, the authors have assumed that the distribution and sizes of particles in the batch are random, but the particle-size distributions are known.

The metaball modelling with metallic porous materials containing diffusion bridges could be suitable for other sintered materials, for example Cu, Cu alloys Ni, and Ni alloys, including metal-ceramics composites, etc. The produced materials have relatively high porosity. The porosity values obtained are comparable to other data presented in [8,11,30,31]. Depending on the planned uses of the porous material, it is necessary to obtain a suitable size of voids. In pore surface chemistry [40,41], there is relevant information about the size distribution of voids in the range of micro- and mesopores. The obtained adsorption isotherms (Figure 3) show a lack of porosity in the ranges of micro and meso. However, the porosity in the macro-pore range is important when the porous material is applied for construction purposes as reported in [1,2,3,4].

The isotherms (Figure 3), obtained according to IUPAC classification (International Union of Pure and Applied Chemistry), are placed in second class with slight hysteresis. The results demonstrate non-porous or macroporous materials and the majority of particles making the porous material have a area size of over 500 nm. The pore volume *V*_t_ is probably caused by this fact.

## 4. Materials and Methods

Metallic precursor composition is made of iron-based powders: Distaloy SE, ASC 100.29 and Fe_2_O_3_ powder. Cu is used as a reaction catalyst. The powders are mixed with [Cu] copper (diffusion catalyst) and [Fe_2_O_3_] iron (III) oxide (foaming agent—space holder) in the form of powder. The prepared specimens are not internally compacted. They are affected only by external gravity reactions. The shape of ceramic conical dies determines the dimensions of the sintered samples; they are max. 14 mm high and 16 mm in diameter. The properties of iron-based materials are shown in Table 2.

In Table 3 the total percentage content of materials forming powder is shown.

**Figure 11:**
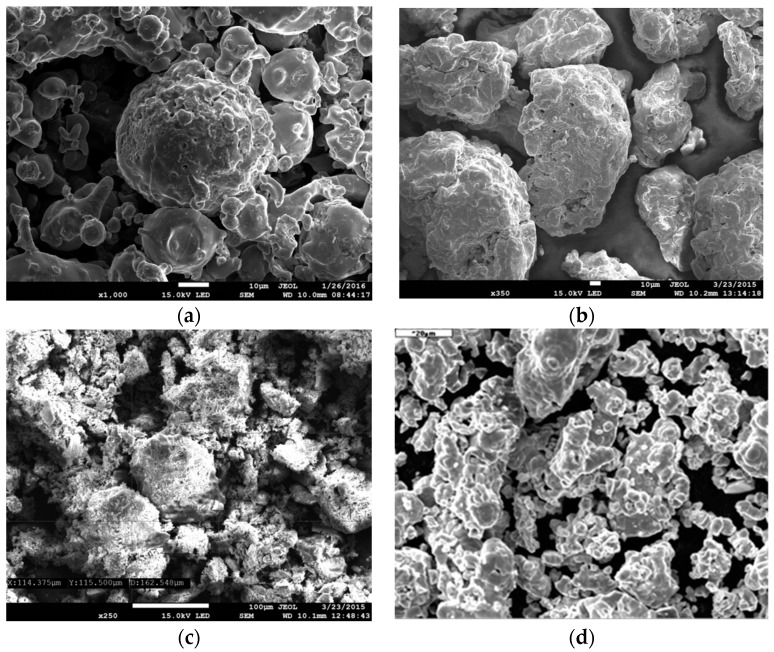
SEM imaging of applied powders: (**a**) ASC 100.29; (**b**) Distaloy SE; (**c**) iron (III) oxide; (**d**) copper powder.

To observe the dimensions and shape of the particles in all applied powders (Table 2), scanning electron microscopy (JSM-7100F, JEOL Musashino, Akishima, Tokyo, Japan) was carried out. Figure 11 shows the results.

The specimens were sintered at a temperature of 1130 °C using tube furnace. Oxides reducer gas—hydrogen—is obtained in the process of ammonia dissociation: 2NH_3_ ↔ N_2_ + 3H_2_. After 50 min of sintering, the samples were moved to the cooling zone. High temperature and hydrogen gas affected iron (III) oxide reduction. Microscopic observation shows that the hollow space inside the solid structure appeared. The reduction of iron (III) oxide caused the formation of porosity. Particles of iron base powder were connected on their edges creating diffusion bridges. At a temperature of 1085 °C copper powder changed from solid to liquid phase. The melted copper was the reaction catalyst.

Nitrogen adsorption isotherms were determined by Micromeritics ASAP 2020 volumetric adsorption analyser (Micromeritics Instrument Corporation, Norcross, GA, USA) at a temperature of −196 °C. All the samples were degassed for 2 h at 200 °C prior to the measurement. On the basis of low-temperature nitrogen adsorption isotherms obtained experimentally the basic parameters characterizing the structure of the test samples were determined. The specific surface area BET (*S*_BET_) [41,42] was calculated on the basis of the nitrogen adsorption isotherm in the range of relative pressures between 0.05 and 0.2. The total pore volume was calculated using a single point on the adsorption isotherm for the relative pressure *p*/*p*_0_ = 0.99 which probably resulted in pore volume *V*_t_. The total percentage contents of applied materials are shown in the Table 4.

### Modelling of the Metallic Porous Materials

As mentioned above, to model porous materials containing diffusion bridges a specialized library generating objects of metaball type was chosen.

Metaballs are commonly used in computer graphics for simulating organic objects. This type of objects can be of different shapes, but the most commonly used shape is a sphere. The metaball is characterized by its influence field. The field of the object can be described by the following formula:

(1) $$f_{m}(x,y,z)=\frac{R^{2}}{{\left(x-x_{0}\right)}^{2}+{\left(y-y_{0}\right)}^{2}+{\left(z-z_{0}\right)}^{2}}$$

where *R* is the radius of the meatball and (*x*_0_, *y*_0_, *z*_0_) is the centre.

To visualize an object, formed of many metaballs, the fields of all particles should be added. To define the surface of created particle a thresholding value should also be chosen. The surface of the created object is defined by the isosurface for which the value of the field is equal to the threshold *T*. The volume of the object consists of all values greater than the threshold *T*.

(2) $$T=\sum_{i=1}^{n}f_{mi}(x,y,z)$$

To generate the structures of diffusion bridges by using the metaball objects the Blender program was used.

The process of porous materials generation consists of following steps:

- generation of the iron and oxide particles with random size and distribution based on sieve analysis
- simulation of mixing and filling a container using physical modelling
- removal of oxide balls, exchanging iron balls by metaballs of the correct size
- modelling of sintering by setting proper threshold value

The sample model of the structure corresponding to the actual structure of the bridge (Figure 6b) is shown in Figure 7.

## 5. Conclusions

This article deals with the modelling of metallic porous materials containing diffusion bridges produced by the sintering of metal powders. Because of the stochastic distribution of the particles making up the obtained sinters, the mathematical description of the metallic porous materials is very complex. The presented modelling method and simulation results prove that it is possible (using metaball objects implemented in the Blender environment) to graphically show the morphological structure of spatial configurations that are close to the microstructure obtained by means of optical microscopy.

The demonstrated modelling method works for all cases where one is able to model the shape of the particles and the distribution of their size. In the described case, the authors modelled structures made of particles of possibly regular shapes, which are characteristic of metallic powders produced by spraying. In order to obtain a good match between the modelled structure and the real metallic porous structure, one has to conduct a sieve analysis to determine the distribution of the particles’ size and a metallographic examination determining the morphology of the powder particles.

In a subsequent stage of the modelling of diffusion bridges produced by sintering of metal powders, the authors plan to use data obtained from µCT, which will enable them to produce a more accurate model, reflecting the spatial distribution of the particles occurring in the actual structure. Such a model will enable one to conduct strength analyses for various structures of metallic porous materials.

## Figures and Tables

**Table 1:** Sieve analysis—percentage of different grain sizes.

Grain Structure (μm)	Sieve Analysis	
	ASC 100.29 (%)	Distaloy SE (%)
<45	18	22
45–150	65	76
150–180	17	2
>180	0	0

**Table 2:** Chemical composition and average particle size of iron base powders.

Powder	Chemical Composition (Mas. %)					Average Particle Size (μm)
	C	Cu	Ni	Mo	Fe	
ASC 100.29	<0.01	-	-	-	Balanced	45–180
Distaloy SE		1.5	4	0.5	balanced	45–180

**Table 3:** Total percentage content of applied materials.

Specimens	Composition (%)			
	ASC 100.29	Distaloy SE	Cu	Iron (III) Oxide
ASC 100.29	85	-	5	10
Distaloy SE	-	85	5	10

**Table 4:** Total percentage content of applied materials.

Material	S_BET_ (m^2^/g)	*V*_t_ (cm^3^/g)
ASC 100.29	26.975	0.0267
Distaloy SE	2.187	0.0036
